# Epidemiological and Clinical Features of Pediatric Open Globe Injuries: A Report from Southern Iran

**DOI:** 10.18502/jovr.v18i1.12729

**Published:** 2023-02-21

**Authors:** Ali Azimi, Fardad Abdollahi, Elham Sadeghi, Amir Reza Farsiani, Shadi Moshksar, Maryam Nadi

**Affiliations:** ^1^Poostchi Ophthalmology Research Center, Department of Ophthalmology, School of Medicine, Shiraz University of Medical Sciences, Shiraz, Iran; ^2^Department of Ophthalmology, Zanjan University of Medical Sciences, Zanjan, Iran

**Keywords:** Eye Injury, Ocular Trauma, Pediatric, Open Globe Injury, Epidemiology

## Abstract

**Purpose:**

To evaluate the epidemiological features of open globe injury (OGI) in a tertiary ophthalmic center in the south of Iran.

**Methods:**

The medical files of pediatric patients diagnosed with OGI between March 2014 and March 2019 were reviewed retrospectively. Demographic data, laterality, time of injury, cause of trauma, location and mechanisms, complications, and the involved tissues, visual acuity, type of operation, and antibiotic therapy were all analyzed. Data were processed using the SPSS.

**Results:**

In total, 110 eyes of 108 patients were included. Ages < 7 years comprised 49.1%, 7–12 years 26.4%, and 13–18 years 24.5% of cases. Of the 108 patients, 76 (70.3%) were males. No significant difference between right versus left eyes was seen. The incidence of OGI was lowest in winter and highest in spring, and it had more prevalence on the weekends. Sharp objects were the most common cause of OGI in ages < 7 years, while blunt objects, accidents and falls, and guns and fireworks were more prevalent in older children. Home was the most common place of injury overall. The most common type of injury was penetrating trauma. Upon arrival, most of the children had a visual acuity < 0.1 decimal. Primary wound closure was the most prevalent type of surgery done predominantly within 24 hr from admission time.

**Conclusion:**

Ages < 7 years and male gender were the most common age and sex of pediatric OGI, respectively, and sharp objects were the predominant etiology. Early management and primary repair are essential for prevention of complications such as endophthalmitis and amblyopia.

##  INTRODUCTION

Twenty to fifty percent of all ocular injuries occur in the pediatric age group. Due to a lack of cooperation and poor compliance for assessment and therapy, this group's management is complicated.^[1]^ Pediatric ocular trauma is the most leading cause of acquired unilateral vision loss in childhood, especially in developing countries.^[2]^ Despite all that has been done to reduce the risk of trauma in children, it remains common worldwide.^[3]^

Ocular injuries are divided into two main groups: closed globe injuries and open globe injuries (OGIs). An OGI is a severe form of trauma leading to a full-thickness defect in the cornea, sclera, or both, exposing the intraocular compartments to the external environment. OGIs are classified into four groups: penetrating injury (only an entrance wound or same entrance/exit wound), perforating injury (separate entrance and exit wounds), rupture (resulting from blunt trauma causing a full-thickness defect at the weakest point of the eyewall), and intraocular foreign body (IOFB). The lack of treatment in childhood trauma may lead to various complications such as cataract, retinal detachment, vitreous hemorrhage, corneal opacity, amblyopia, IOFB and toxicity due to chronic foreign bodies, endophthalmitis, and sympathetic ophthalmia.^[4]^

Knowledge of the epidemiological characteristics of the OGI can assist in the prevention of catastrophic damage to children's physiological and psychological health. World Health Organization (WHO) has recognized childhood blindness as one of the leading causes of preventable blindness.

Determination of epidemiologic risk factors and prognosis of OGIs are essential in achieving a healthier outcome and also reducing its prevalence.^[5]^ Therefore, we carried out a retrospective study to evaluate the clinical course and outcomes in all patients younger than 18 years old who were admitted to this tertiary referral university-affiliated ophthalmology center in the south of Iran due to OGI.

##  METHODS

In this retrospective study, the medical charts of pediatric patients suffering from OGIs were reviewed. The medical records of 110 eyes of 108 children (age ≤ 18 years) admitted to this hospital due to ocular trauma and diagnosed by slit-lamp examination or examination under anesthesia from March 2014 to March 2019 were evaluated. Patients who had a full-thickness ocular injury repaired at other centers were excluded.

In the medical charts, the initial ophthalmology examination sheets, hospital records, details of the operation notes, and outpatient follow-up were all reviewed. Demographic data were collected on age, sex, injured eye, ocular status before the trauma, laterality, place of trauma, month and year of injury, and mechanism of trauma.

The initial best corrected distance visual acuity (BCDVA), evaluated by Snellen chart, was recorded. No light perception (NLP) visual acuity (VA) was confirmed using an indirect ophthalmoscope with a bright and highest intensity light source. Clinical data such as intraocular pressure (IOP) with Goldman tonometer, location of injury (home, school, and street), type of injury, uvea and pupil status, hyphema, lens status, vitreous, retina and choroidal conditions, involvement of orbit, presence of uveitis or endophthalmitis, and types of required surgeries were all recorded. Based on the Birmingham Eye Trauma Terminology (BETT), cases were classified into penetrating, perforating, or IOFB injury.

The study protocol was approved by the ethics committee of the university hospital. It adhered to the tenants of the Declaration of Helsinki.

Continuous parameters were reported as mean ± SD. Chi-square and Fisher's exact tests were used to compare the categorical data, and independent *t*-test and ANOVA tests were used to compare the continuous data. Statistical analysis was performed using the SPSS for Windows (SPSS Inc, Chicago, IL). Data analysis was interpreted using a significance level of *P* < 0.05.

##  RESULTS

A total of 110 eyes of 108 pediatric patients initially diagnosed as OGI was included in this five-year study period (from March 2014 to March 2019). Patients' mean age was 7.8 ± 5.2 years (range, 6 months to 18 years) with a median of 7 years and a mode of 2 years. The mean age was 8.7 ± 5.4 years for boys, while the girls' mean age was 5.8 ± 4.1 years (*P* = 0.006).

Patients' eyes were divided into three different age groups: < 7 years (49.1% *n* = 54), 7–12 years (26.4% *n* = 29), and 13–18 years (24.5% *n* = 27). The majority of our cases were in the preschool age ( < 7years) group (*P* < 0.001).

In our study, 69.1% of eyes were related to the male gender (*n* = 76) and 30.9% were related to females (*n* = 34). Boys were statistically more susceptible to experience OGI than girls (ratio 2.2:1; *P* < 0.001). This predominance rose sharply from preschool children to older children 13–18 years old (1.57:1 to 8:1) (*P* < 0.001).

**Figure 1:**
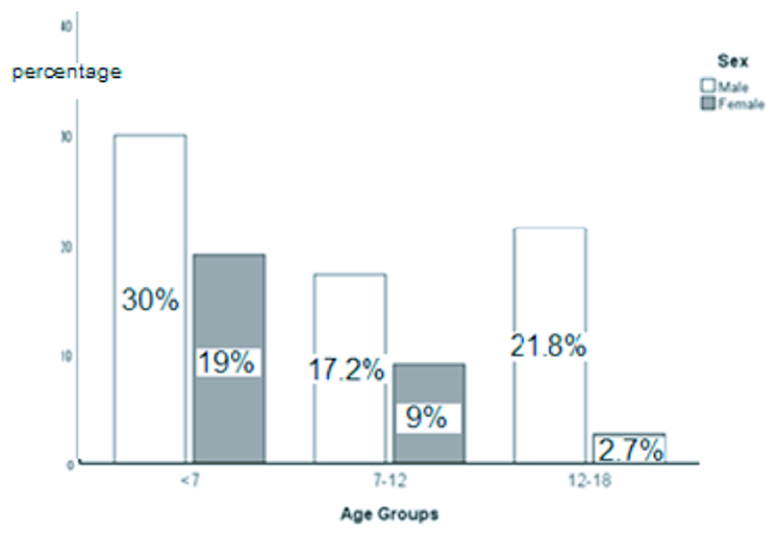
Distribution of trauma according to sex and age groups.

As shown in Figure 1, there is a negative correlation between age and incidence of OGI in girls.

**Figure 2:**
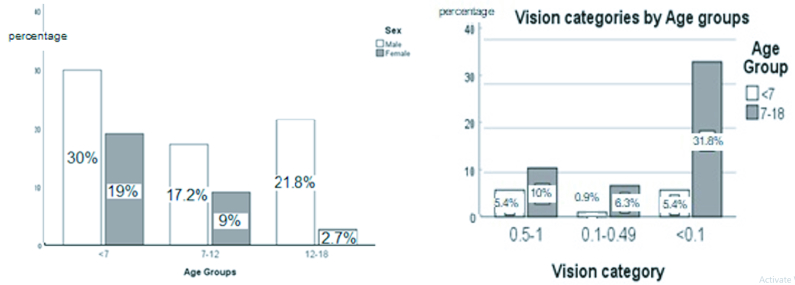
Vision categories by sex and age groups.

**Table 1:** Number of OGI by season.


orange**Season**	orange**Number**	orange**Percentage**	
Spring	34	30.9	*P* = 0.76
Summer	28	25.5	
Fall	25	22.7	
Winter	23	20.9	
	
	
white<bcol>4</ecol>OGI, open globe injury

**Table 2:** Distribution of trauma by days of the week.


orange**Days of the week**	orange ***N***	orange**Percentage**	
Saturday	14	12.7	*P* = 0.37
Sunday	18	16.4	
Monday	15	13.6	
Tuesday	12	10.9	
Wednesday	11	10.0	
Thursday	19	17.3	
Friday	21	19.1	
	
	

**Table 3:** Causative objects of open globe injuries in children.


orange**Objects**	orange ***N*** ** (%)**	orange**Objects**	orange ***N*** ** (%)**
Knife	23 (20.9%)	Belt	3 (2.7%)
Accidents	16 (14.5%)	Eye glass	3 (2.7%)
Wood	12 (10.9%)	Fall	3 (2.7%)
Metal, wire, nails	8 (7.2%)	Fist	3 (2.7%)
Stone	7 (6.4%)	Animal horn	2 (1.8%)
Gun	5 (4.5%)	Pen	2 (1.8%)
Fireworks	5 (4.5%)	Explosion	1 (0.9%)
Needle	4 (3.6%)	Others	13 (11.8%)
	
	

**Table 4:** Place of injury according to age groups.


orange**Age groups (yr)**	orange **Place of injury**			
	orange**Home**	orange**School**	orange**Outdoor**	orange ***P*** **-value**
	orange ***N*** ** (%)**			
< 7	43 (39.1)	1 (0.9)	10 (9.1)	< 0.001
7–18	17 (15.5)	12 (10.9)	27 (24.5)	< 0.001
	
	

**Table 5:** Complications and eye layers involvement.


orange**Complication**	orange ***N*** ** (%)**	orange**Complication**	orange ***N*** ** (%)**
Corneal laceration	59 (53)	Retina: hemorrhage	5 (4.5)
Scleral laceration	55 (50)	Retina: edema	3 (2.7)
Uvea in wound	5 (4.5)	Retina: tear	2 (1.8)
Hyphema	76 (69)	Retina: dialysis/detachment	3 (2.7)
Iris injury	28 (25)	External muscles	2 (1.8)
Hypotony	108 (98.18)	Lid	19 (17.2)
Optic nerve injury	26 (23)	Lacrimal system	2 (1.8)
Lens: cataract	40 (36)	Orbit: fracture	5 (4.5)
Lens: subluxated/dislocated	3 (2.7)	Orbit: foreign body	1 (0.9)
Vitreous: hemorrhage	17 (15.4)	Orbit: hemorrhage	4 (3.6)
Vitreous: prolapse	2 (1.8)	Inflammation: uveitis	9 (8.1)
Choroid: hemorrhage	2 (1.8)	Inflammation: endophthalmitis	5 (4.5)
Choroid: rupture	1 (0.9)	
	
	

**Table 6:** Operations after the initial surgeries.


orange**Operation**	orange ***N*** ** (%)**	orange**Operation**	orange ***N*** ** (%)**
Lensectomy	21 (19)	Pupiloplasty	4 (3.6)
Deep Viterectomy	21 (19)	Phaco	2 (1.8)
Anterior Viterectomy	2 (1.8)	Tarsoplasty	2 (1.8)
Suture removal	9 (8.1)	Iris cystectomy	2 (1.8)
Enucleation	8 (7.2)	Lateral canaloplasty	1 (0.9)
IOFB removal	6( 5.4)	Dacryocystorhinostomy	1 (0.9)
IOL implantation	5 (4.5)	ERMs removal	1 (0.9)
PCIOL implantation	3 (2.7)	Posterior capsulectomy	1 (0.9)
Lid reconstruction	4 (3.6)	Orbital wall repair	1 (0.9)
	
	

**Table 7:** Route of antibiotic injection by age groups.


orange**Age groups (yr)**	orange **Route of antibiotic**			orange ***P*** **-value**
	orange**Intravitreal**	orange**Intravenous**	orange**Both**	orange**0.008**
	orange ***N*** ** (%)**			
< 7	11 (11.3)	27 (27.8)	12 (12.4)	
7–18	3 (3.1)	39 (40.2)	5 (5.2)	
	
	

The rate of right eye involvement was 53.6% (*n* = 59), and the left eye was 46.3% (*n* = 51) with no statistically significant difference. There were two cases with bilateral eye involvement caused by motor vehicle accident and mine explosion.

As shown in Table 1, the incidence of OGI was lowest in winter and highest in spring with no statistically significant difference by season (*P* = 0.76). Incidences were more on the weekends, but there were no significant differences regarding the day of the week (weekdays vs weekends; *P* = 0.37) [Table 2].

Most of the injuries were caused by sharp objects (37.3% *n* = 41), followed by blunt objects (35.3% *n* = 39), accidents and falls (17.3% *n* = 19), and guns and fireworks (10% *n* = 11). A statistically significant difference was identified between causative objects of the injuries and different age groups (*P* = 0.027). Sharp objects comprised a greater number of OGI in the preschool age group ( < 7 years), while blunt objects, accidents and falls, and guns and fireworks accounted for more injuries in older children (7–18 years). Knives and wooden sticks were the most common tools among the sharp and blunt objects that provoked OGI in all children [Table 3].

Home was the most predominant place of injury (54% *n* = 60), followed by outdoor environment (street 25.5% *n* = 28, farm 7.3% *n* = 8, industrial places 0.9% *n* = 1) and school (11.8% *n* = 13). Home was the top place that injuries occurred amid the preschool age group ( < 7), while the outdoor environment was more frequent in older ages (All *P*s < 0.001) [Table 4].

Most of the injuries (68% *n* = 75) occurred while playing. In decreasing order of frequency, accidents (17.3% *n* = 19), assaults (10% *n* = 11), and occupation-related factors (4.5% *n* = 5) were the other activities that led to injuries (*P* < 0.001).

The majority of the injuries in both boys and girls happened during playing, but assaults and occupation-related factors encompassed a higher percentage of injuries in boys (boys to girls' ratio for assaults is 7.3:2.7, and for occupational is 3.6:0.9) (P < 0.001).

The most common types of injury, according to BETT, were penetrating injuries (60.9% *n* = 67), followed by rupture (7.3% *n* = 8), IOFB (6.4% *n* = 7) and perforating injury (0.9% *n* = 1). Of the 110 traumatized eyes, 25 (22.7%) had multiple eye involvement and could not be categorized based on the BETT system. Two (1.8%) cases only had a partial-thickness laceration of the eye.

Penetrating traumas were the primary type of injuries in all age groups and genders, however, rupture injuries were observed mainly in boys and children over seven years of age (87%, seven out of eight rupture cases). We had only one case of perforating injury; a 16-year-old boy hit by a shotgun while playing on the farm.

Laceration layers in penetrating injuries were mostly corneoscleral (47.7% *n* = 32), followed by corneal (29.8% *n* = 20) and scleral (22.3% *n* = 15) layers. Corneoscleral involvement rose sharply in boys as compared to girls (25:10 cases). There were no significant statistical differences in penetrating injuries between different genders (*P* = 0.38) and age (*P* = 0.16) according to the laceration layers.

The mean size of lacerations in corneal penetrating injuries was 5.7 ± 3.1 mm with a median of 5 mm, while it was 3.47 ± 2.3 mm in scleral penetrating injuries with a median of 3 mm.

In 57 cases of corneal penetrating injuries, the main site of laceration was nasal (35% *n* = 20), followed by temporal (28% *n* = 16), central (15.8% *n* = 9), superior (12.3% *n* = 7), and inferior (8.8% *n* = 5).

In 52 cases of scleral penetrating injuries, the most common site of laceration was nasal (36% *n* = 19), followed by temporal (28% *n* = 15), inferior (17% *n* = 9), superior (15% *n* = 8), and central (1.9% *n* = 1) [Table 5].

Upon arrival, VA could not be evaluated in 36.4% (*n* = 40) of the cases because they were uncooperative. In four cases, VA was recorded using the CSM method (central, steady, and maintained eye position), which all of them presented C+S+M+VA.

BCDVA upon arrival was categorized into three groups: 0.5–1, 0.1–0.49, and under 0.1 decimal. In 66 eyes, which had registered VA upon arrival, most of the eyes had VA under 0.1 (62.1% *n* = 41), followed by 0.5–1 (25.7% *n* = 17) and 0.1–0.49 (12.1% *n* = 8).

In under 0.1 categories, VA was counting fingers in 13 cases, hand-motion in 6 cases, light perception in 9 cases, and NLP in 13 cases. As shown in Figure 2, in the male group, the VA under 0.1 was clearly higher than the female group. Additionally, the cases between 7 and 18 years old, compared to patients < 7 years old, significantly presented the VA under 0.1 upon arrival.

Operation was performed on 93.6% of the cases (*n* = 103) with an initial diagnosis of OGI. Ninety-five percent of the operations (*n* = 98) were performed within 24 hr from the admission time. Five children only had an examination under sedation (EUS). Three children were discharged voluntarily despite the physicians' recommendation for surgery.

Out of the 98 patients who underwent surgery, 47.3% were operated on once; 33.6% (*n* = 37) had two surgeries; and 12.7% (*n* = 14) had more than two surgeries. Primary repair of lacerations was the most common type of the initial operation (75.7% *n* = 78). Peritomy comprised 24.2 % (*n* = 25) of the initial operations. Table 6 shows operations that were done after the initial surgeries. Of the 110 eyes with OGI, injuries finally led to the enucleation of the eyes in 7.2% of the cases (*n* = 8).

About 88% of the patients received antibiotics during admission to the hospital. Children in preschool-age received more intravitreal antibiotics or a combination of both intravitreal and intravenous antibiotics, while antibiotic therapy in older aged children was mainly via the intravenous route (*P* = 0.008) [Table 7]. About 84% of the patients (*n* = 93) received intravenous antibiotics. The mean days of receiving antibiotics were 3.4 ± 1.2 days, with a median of three days. A combination of ceftazidime and vancomycin were the most common antibiotics used intravenously (75% *n* = 70), followed by cefazolin and gentamicin (22% *n* = 21) and ceftriaxone and vancomycin (2.1% *n* = 2). From 20 cases who received intravitreal antibiotics during operation, cefazoline was the main choice (18 out of 20), and two other cases received intravitreal gentamycin and imipenem.

##  DISCUSSION

Pediatric OGI can cause lifelong complications and unilateral blindness.^[6]^ Since prevention is better than cure, solutions must be found to reduce it. Our study revealed an increase in OGI in preschool-aged children ( < 7 years). This is similar to the study by El-Sebaity et al,^[7]^ but some reports have found increasing OGI risk in the 7–12 years old age group.[8–10]

Different studies showed different ratios of female OGL, but all agree that males' injury is statistically higher than females' injury, which is related to the propensity of the male gender to higher risk activities with less parental supervision as part of their natural growth.[4, 11–15] Besides, the differences between the sexes among the older age groups (12–18 years) increased significantly, and there was a negative correlation between age and incidence of OGI in females. It suggests that girls' dangerous and risky activities may reduce with aging.

There was no significant difference between the right and the left eyes. This is similar to the results of the study by Tan et al,^[16]^ however, some reports have revealed that in adult cases, the right eye is more susceptible to injuries because, in adults, most injuries occur in the workplace.[17, 18] It must also be kept in mind that severe traumatic accidents, explosions, or intentional assault injuries may induce bilateral ocular involvement.

We have reported the OGI trend during vacation, as mentioned in other studies.[19, 20] A higher incidence of OGI on Fridays shows that children have more dangerous activities on the weekend. The risk increase in spring is a result of Norouz holidays in Iranian culture. At this time, the weather becomes warmer, and children tend to play outdoor group games without parental supervision. It may be avoided with parental supervision, keeping dangerous devices out of children's reach, and educating children about proper safety considerations.

The prevailing etiological agent causing OGI in the preschool age group is sharp material such as knives, which correlates with previous studies.[6, 21, 22] Ocular trauma with knives usually happens accidentally. It is crucial to either decrease access to sharp objects or replace them with round blunt-tipped knives.^[1]^ Older children are injured more by blunt objects, accidental falls, guns, and fireworks, predominantly in males related to practicing more aggressive behavior. These injuries can be prevented with proper training and using protective eyewear. Ocular trauma during car and motor accidents were the cause of 14.5% of OGI, and it is usually due to glass particles or blunt trauma. Using seatbelts and helmets may reduce the risk of experiencing ocular trauma during a road accident.

Prior studies have revealed that ocular injuries most frequently occur at home.[4, 12, 23] In this study, home was the main place for the incidence of ocular injuries occurring in preschool-aged children. As this age group spends most of their time at home, the use of toys with blunt edges and increased parental supervision may reduce the ocular trauma risk. Covering the sharp edges of household items with protective equipment may also be useful. Further awareness of parents and babysitters is recommended to prevent preschool-age OGI.^[24]^ Some reports have shown that outdoor spaces are the most common place for OGI occurrences in pediatrics.[25, 26] In our study, the outdoor environment is the primary place for older children, especially in the male group, due to accidents, assaults, and occupation-related factors. Educating children to follow safety principles and practice anger management can be useful in reducing the occurrence of accidents, assaults, and occupational hazards that may lead to OGI.

Analysis of OGI in this report revealed that penetrating injuries were the most common type of ocular trauma (60.9%), which was consistent with data published by Puodžiuvienė et al.^[8]^ The rate of globe ruptures was 7.3%, which was lower than the data reported by Court et al.^[27]^ OGI with IOFB is more often experienced in adults. However, it is not rare in children.^[28]^ The rate of IOFB injuries in our study was 6.4%. Compared to other types of OGI in pediatrics, perforating injury is not common in this group^[29]^ as this type of injury is most often caused by shotgun usage which is not normally used in this age group. In our study, one patient was 16 years old with perforating OGI caused by firing a shotgun while playing on the farm.

The majority of wounds involve both cornea and sclera (47.3%), and hypotony is the most common presentation (98.18%). Two patients did not have hypotony because the laceration was partial thickness. Hyphema was the second most common sign (69%). One study had shown that hyphema was significantly related to closed globe injuries.^[30]^ The other prevalent signs in OGI are traumatic cataract, iris injury, optic nerve injury, lid laceration, and vitreous hemorrhage.

In this study, 37.3% of cases had < 0.1 VA upon arrival where 78% were males. This measurement is justified as more severe injuries occur in males due to their inherently more aggressive behavior.

Primary repair of the wound and repositioning of the prolapsed tissue is the most common surgery performed initially, usually within 24 hr from admission.^[4]^ Endophthalmitis is one of the most serious and poor prognosis complications after OGI, which is preventable by primary wound closure.^[31]^ About one-third (33.6%) of cases underwent surgeries twice, and 12.7% of them underwent surgeries three times. Lensectomy and deep vitrectomy were the most common types of surgery performed after the initial operation. In an effort to prevent sympathetic ophthalmia, 7.2% of cases underwent enucleation and conformer placement surgery due to devastating injury and NLP VA.

Antibiotics administration plays a role in prophylaxis and treatment of endophthalmitis.^[32]^ Traumatic endophthalmitis is usually seen in delayed wound closure, IOFB, posterior capsule rupture, delayed initiation of prophylactic antibiotic therapy after 24 hr from ocular trauma, and wound contamination with organic material.[33, 34] The antibiotic selected for treatment should have a broad spectrum acting against a larger group of microorganisms.^[35]^

The visual prognosis in pediatric OGI is not good. Statistics show that patients with ocular trauma in one eye are susceptible to trauma in the fellow eye.^[36]^ Assessing ocular trauma is more important in the pediatric group due to longer lifespans and the more incidence of ocular complications in this group including cataract, retinal detachment, vitreous hemorrhage, corneal opacity, amblyopia, IOFB and toxicity due to chronic foreign bodies, endophthalmitis, and sympathetic ophthalmia; hence emphasizing that prevention is better than treatment. Education of parents, babysitters, and school teachers about children's supervision and choosing suitable toys according to child's age is needed. Keeping dangerous objects with sharp edges out of the reach of children is essential. Using protective eye glasses may play a useful role in preventing ocular trauma, while playing or working with sharp objects. To prevent eye injuries during an accident, using safety measures like suitable child seats, seat belts, and helmets are effective. Older children should be educated to avoid using guns, fireworks, and explosive devices.

This report is a retrospective and non-randomized study with some limitations because it was limited to medical files during hospitalization. Further studies may be needed to determine the overall burden of disease, post-discharge follow-up data, final VA, and delayed complications.

The second limitation is that as ocular trauma patients were referred to multiple centers, the actual number of patients with OGI recorded in this report is incomplete.

In summary, the results suggest the importance of prevention in reducing the frequency of ocular trauma in children due to longer lifespan. Also, it has recommended early primary wound closure to reduce or prevent devastating ocular complications. Additionally, it has counsel that extended follow-up is necessary to reduce and manage further complications such as amblyopia. More studies are recommended to accurately evaluate the prognosis of OGI in the long-term.

##  Financial Support and Sponsorship 

None.

##  Conflicts of Interest 

The authors declare that they have no conflict of interest.
